# The First Trimester Gravid Serum Regulates Procalcitonin Expression in Human Macrophages Skewing Their Phenotype *In Vitro*

**DOI:** 10.1155/2014/248963

**Published:** 2014-03-05

**Authors:** Damiano Rami, Martina La Bianca, Chiara Agostinis, Giorgio Zauli, Oriano Radillo, Roberta Bulla

**Affiliations:** ^1^Department of Life Sciences, University of Trieste, Via Valerio 28, 34127 Trieste, Italy; ^2^Institute for Maternal and Child Health, IRCCS “Burlo Garofolo”, 34137 Trieste, Italy

## Abstract

Procalcitonin (PCT) is one of the best diagnostic and prognostic markers in clinical practice, widely used to evaluate the evolution of bacterial infections. Although it is mainly produced by thyroid, during sepsis almost all the peripheral tissues are involved in PCT production. Parenchymal cells have been suggested as the main source of PCT expression; however the contribution of macrophages is not clear yet. In response to environmental cues, tissue macrophages acquire distinct functional phenotypes, ranging from proinflammatory (M1) to anti-inflammatory (M2) phenotype. Macrophages at the fetal-maternal interface show immunosuppressive M2-like activities required for the maintenance of immunological homeostasis during pregnancy. This study aims to clarify the ability to synthesise PCT of fully differentiated (M0), polarized (M1/M2) macrophages and those cultured either in the presence of first trimester gravid serum (GS) or pregnancy hormones. We found out that M1 macrophages upregulate PCT expression following LPS stimulation compared to M0 and M2. The GS downregulates PCT expression in macrophages, skewing them towards an M2-like phenotype. This effect seems only partially mediated by the hormonal *milieu*. Our findings strengthen the key role of macrophages in counteracting inflammatory stimuli during pregnancy, suggesting PCT as a possible new marker of M1-like macrophages.

## 1. Introduction

Procalcitonin (PCT) is the prohormone of calcitonin (CT) and is composed of 116 amino acids with a molecular mass of about 14 kDa [1]. PCT is usually released into the circulation from neuroendocrine cells of the thyroid gland (parafollicular or C cells) [2] and the lungs (K cells) [3], reaching physiological concentrations lower than 0.05 *μ*g/L in the plasma of healthy people [4]. During an inflammatory status, PCT derives from almost all cell types and tissues, including monocytes and parenchymal tissues, making its upregulation less dependent on one type of cell, tissue, or organ [5–8]. The synthesis of PCT is mainly stimulated through bacterial endotoxins lipopolysaccharides (LPS) [9] and, to lesser extent, from the proinflammatory cytokines IL-1*β*, IL-6, and TNF*α* [10, 11]. Its levels are however attenuated by the release of IFN*γ* in response to viral infections [12]. PCT is now generally accepted as a good prognostic and diagnostic marker of bacterial infection, due to its high stability in serum and its ability to respond more rapidly to inflammatory stimuli than other laboratory parameters of infection, such as C-reactive protein (CRP) and TNF*α*, making it particularly suitable for routine laboratory analysis [13]. Macrophages are an essential component of innate immunity and play a central role in inflammation and host defense [14, 15]. In response to environmental cues, mononuclear phagocytes acquire distinct functional phenotypes, ranging from a “classical” proinflammatory/antiangiogenic (M1) to an “alternatively” anti-inflammatory/proangiogenic (M2) phenotype, which represents extremes of a continuum in a universe of different activation states [16, 17]. Diversity, plasticity, and flexibility are hallmarks of cells of the monocytes-macrophages lineage, either under physiological or pathological conditions. Therefore, the functional skewing of macrophages is one of the best mechanisms to modulate both the systemic and local immunity [18]. Macrophages play a key role in counteracting inflammation during pregnancy [19]. This state represents an intriguing immunological condition, in which an immunosurveillance and a repression of the immune system both coexist, thanks to which the mother is able to tolerate the presence of the foetus and to quickly respond to local and systemic inflammatory stimuli [20]. Up to now, there are several studies in the literature which show freshly isolated human monocytes expressing PCT [8, 11, 21]; on the other hand, the contribution of fully differentiated and polarized macrophages in PCT expression is not known yet. In this study we investigate the involvement of primary human macrophages in PCT production, evaluating the modulation of PCT expression in these immune cells during normal and inflammatory conditions; besides, we aim to show if the synthesis of this prohormone can be somehow regulated during the macrophage polarization towards M1 and M2 phenotypes. Moreover, based on the importance of counteracting inflammation during gestation, our study examines how the human serum of the first trimester of pregnancy (GS) modulates PCT expression in fully differentiated and polarized macrophages, analysing its capability to skew their phenotype *in vitro*. For the first time, we showed that PCT is expressed both at gene and protein level in fully differentiated macrophages, and it is upregulated in those with M1 phenotype under stimulation with the proinflammatory stimulus LPS. Moreover, the GS has the capability to downregulate PCT expression in human macrophages* in vitro*, skewing them into an M2-like phenotype.

## 2. Materials and Methods

### 2.1. Reagents and Antibodies

The human cytokines IL-4 and IFN*γ* were purchased from ImmunoTools GmbH (Friesoythe, Germany) and IL-10 from PeproTech EC Ltd. (DBA Italia S.r.l, Milan, Italy). Ultrapure lipopolysaccharide (LPS) from *E. coli* 0111:B4 strain was obtained from InvivoGen (Labogen S.r.l., Milan, Italy). Polyclonal rabbit anti-human PCT was purchased from Abcam (ProdottiGianni, Milan, Italy). CD68 (Macrosialin, mouse anti-human monoclonal antibody, and clone EMB11) and CD206 (Macrophage mannose receptor 1, mouse anti-human monoclonal antibody, and clone MCA2155) antibodies were purchased, respectively, from Dako (Milan, Italy) and AbD Serotec (Milan, Italy). Secondary goat anti-mouse-Cy3 and streptavidin Cy2-conjugated were purchased from Jackson ImmunoResearch (LiStarFish, Milan, Italy); biotinylated swine anti-rabbit IgG were from Dako. The first trimester gravid serum (GS) was obtained at gestational week 12 (normal pregnant women). A pool of fresh sera from healthy blood donors was used as normal human serum (NHS). The sera were heat-inactivated at 56°C for 30 minutes prior to use. An informed consent was obtained from all women participating in the study. The study was approved by the Bioethical Committee of IRCCS, Burlo Garofolo, Trieste, Italy. All the samples were immediately centrifuged, aliquoted, and frozen at −80°C.

### 2.2. Isolation and Differentiation of Human Peripheral Monocytes into Macrophages and Their *In Vitro* Polarization

Peripheral blood mononuclear cells (PBMCs) from healthy blood donors were isolated from anonymous buffy coats, kindly provided by the local blood bank (Immunotransfusional Department, Maggiore Hospital, Trieste, Italy) using Ficoll-Paque Plus density gradient (GE Healthcare Euroclone, Milan, Italy). Residual T and B cells were removed from the monocyte fraction by plastic adherence, after incubation for 2 hours at 37°C and 5% CO_2_ in RPMI-1640 GlutaMAX (Life Technologies, Milan, Italy) supplemented with 10% of NHS and 1% Penicillin/Streptomycin (Sigma-Aldrich). Fully differentiated and M1- and M2-polarized macrophages were obtained by culturing 10^6^ monocytes/mL for 7 days at 37°C and 5% CO_2_ with the same medium above described and replaced twice a week. M1 cells were polarized by stimulating overnight (O/N) with LPS (100 ng/mL) (InvivoGen) and IFN*γ* (500 U/mL) (ImmunoTools), while M2 macrophages were polarized by stimulating O/N with IL-4 (20 ng/mL) (ImmunoTools) and IL-10 (50 ng/mL) (PeproTech) [22, 23].

### 2.3. Stimulation of Macrophages with First Trimester Gravid Serum

To avoid the presence of cellular debris, the first trimester gravid serum (GS) was centrifuged at 10.000 g for 5 minutes at room temperature (RT) and then filtered with Costar Spin-X Centrifuge Tube Filters, 0.22 *μ*m Pore CA Membrane (Corning, Turin, Italy). Fully differentiated macrophages were incubated for 24 hours at 37°C and 5% CO_2_ in RPMI-1640 GlutaMAX (Life Technologies) supplemented with 10% of GS and 1% Penicillin/Streptomycin (Sigma-Aldrich). Cells were then stimulated for 3 hours at 37°C and 5% CO_2_ in the culture medium with 10% NHS and LPS at a final concentration of 100 ng/mL, to induce the gene expression of PCT.

### 2.4. Stimulation of Macrophages with Progesterone, 17*β*-Estradiol, and Human Chorionic Gonadotropin (hCG)

Fully differentiated macrophages were incubated for 24 hours at 37°C and 5% CO_2_ in RPMI-1640 GlutaMAX (Life Technologies) supplemented with 10% of NHS and 1% Penicillin/Streptomycin (Sigma-Aldrich) in the presence of 100 nM of progesterone (PG), 10 nM of 17*β*-Estradiol (E2), and 20 U/mL of human chorionic gonadotropin (hCG), hormones that have been used alone or combined all together. Cells were then stimulated for 3 hours at 37°C and 5% CO_2_ in the culture medium with 10% NHS and 100 ng/mL of LPS. Concentrations of the pregnancy hormones were the same as that reached in the serum of women during the 12th week of pregnancy.

### 2.5. Viability Assay

Cell viability was evaluated by Trypan Blue (Sigma-Aldrich) dye exclusion test. No differences were observed before and after completing the experiments.

### 2.6. RNA Isolation, cDNA Synthesis, and Quantitative Real-Time PCR (qPCR)

RNA was purified from cells with EuroGOLD trifast (Euroclone) according to the manufacturer's instructions. Total RNA was extracted and reverse-transcribed as previously described [24]. Quantitative Real-Time PCR (qPCR) was carried out on a Rotor-Gene 6000 (Corbett, Qiagen, Ancona, Italy) using iQ SYBR Green Supermix (Bio-Rad, Milan, Italy).  Table 1 shows the primer list used for qPCR. The melting curve was recorded between 55°C and 99°C with a hold every 2 s. The relative amount of gene production in each sample was determined by the Comparative Quantification (CQ) method supplied as part of the Rotor Gene 1.7 software (Corbett Research) [25]. The relative amount of each gene was normalized with 18S and expressed as arbitrary units (AU) considering 1 AU obtained from fully differentiated macrophage used as calibrator.

### 2.7. Immunofluorescence

Isolated PBMCs, obtained as previously described, were plated for 48 hours at 37°C and 5% CO_2_ in RPMI-1640 GlutaMAX (Life Technologies) supplemented with 10% of NHS and 1% Penicillin/Streptomycin (Sigma-Aldrich) on 8-chamber culture slides (BD Biosciences Discovery Labware, Milan, Italy) to allow the adhesion of monocytes, which were left to differentiate for 7 days as reported above. Cells were incubated for 24 hours at 37°C and 5% CO_2_ in RPMI-1640 GlutaMAX (Life Technologies) supplemented with 10% of GS and 1% Penicillin/Streptomycin (Sigma-Aldrich) and subsequently stimulated for 3 hours with LPS (100 ng/mL) [8]. Macrophages were fixed and permeabilized with FIX & PERM cell permeabilization kit (Società Italiana Chimici, Rome, Italy) according to the manufacturer's instructions and then blocked with human serum (1 : 60) for 30 min at RT. Afterwards, cells were incubated with primary mAb anti-CD68 (1 : 40) (Dako), mAb anti-CD206 (1 : 50) (Sigma-Aldrich), or rabbit anti-human PCT (Abcam) O/N at 4°C. The binding of CD68 and CD206 antibodies was revealed with goat anti-mouse Cy3-conjugated secondary antibody (1 : 300), while biotinylated swine anti-rabbit (1 : 50) was followed by streptavidin-Cy2 (1 : 200) to detect the anti-PCT polyAb, for 45 minutes in the dark at RT, followed by 4′,6-diamidino-2-phenylindole (DAPI, Sigma-Aldrich) staining. Slides were prepared with *mounting solution *(Dako). Images were acquired with Leica DM3000 microscope (Leica, Wetzlar, Germany) and the pictures were collected using a Leica DFC320 digital camera (Leica).

### 2.8. Quantification of PCT Amount on the Culture Medium of Macrophages

Culture medium of macrophages was taken under sterile condition in a final volume of 150 *μ*L/sample, centrifuged at 12.000 g for 2 minutes at +4°C to avoid the presence of cellular debris, and maintained at −80°C until analysis. The amount of PCT in the culture medium of macrophages was quantified automatically by the Modular Analytics E170 Module (Roche Diagnostics, Milan, Italy) using BRAHMS PCT reagent (Roche, Mannheim, Germany).

## 3. Statistical Analysis

Statistical analysis was performed with Microsoft Office Excel 2003 (Microsoft Corporation, Redmond, CA, USA). Data were reported as mean ± S.E.M. Mann-Whitney test was used to compare two groups of data and *P* value of <0.05 was considered significant.

## 4. Results

### 4.1. Characterization of the Production of PCT in M0 and M1 or M2 Polarized Macrophages

**Figure 1:**
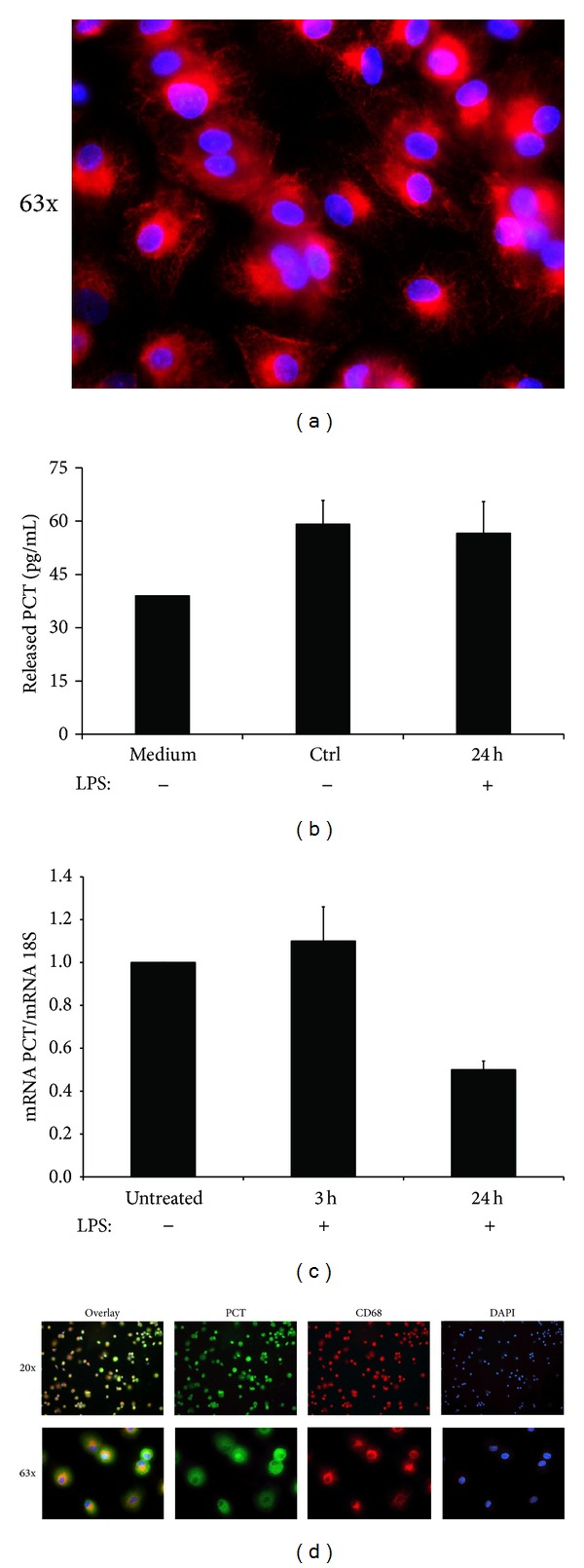
Human macrophages express PCT at mRNA and protein level. (a) Immunofluorescence analysis of human macrophages differentiated from PBMCs. Cells were stained with mAb anti-human CD68. DAPI nuclear staining is shown in blue. Original magnification 63x. (b) Levels of PCT secreted in the cell supernatant by cultured macrophages after 24 hours of stimulation with LPS. Data from 5 independent experiments are shown and no statistical significance has been found between treated and untreated cells. (c) RNA prepared from fully differentiated human macrophages, untreated or stimulated with LPS for 3 and 24 hours, were quantified for PCT and 18S expression by qPCR. Data from 5 independent experiments are shown and represent the mean ± S.E.M. *P < 0.05 (Mann-Whitney test). (d) Double Immunofluorescence analysis of human macrophages for PCT (green) and CD68 (red) expression. Overlay images show the PCT localized throughout the cytoplasm. DAPI nuclear staining is shown in blue. Original magnification 20x (upper panels) and 63x (lower panels). Images were acquired with Leica DM3000 microscope.

We initially investigated the ability of nonpolarized human macrophages (M0) to secrete PCT in the culture supernatant. The purity of human macrophages was confirmed with an immunofluorescence staining against the classical marker CD68 (Figure 1(a)). The levels of PCT were measured in the harvested supernatants of macrophages after 24 hours of culture. As shown in Figure 1(b), unstimulated macrophages produced about 20 pg/mL of PCT and no difference was observed in the supernatants of the cells stimulated with LPS.

The production of PCT by macrophages was also confirmed by the expression of the corresponding mRNA documented by qPCR (Figure 1(c)). The mRNA expression analysis revealed the presence of transcripts for PCT, confirming the synthesis of this protein. In addition, cells stimulated with LPS for 3 hours did not reveal any modulation in mRNA expression. Unexpectedly, 24-hour stimulation with LPS induced a significant downregulation of the transcript for PCT. To further reveal the presence of PCT in human macrophages, a triple staining with DAPI and antibodies against PCT and CD68 was assessed on macrophages cultured in 8-chamber culture slides. PCT was clearly visible and localized throughout the cytoplasm as shown in Figure 1(d).

**Figure 2:**
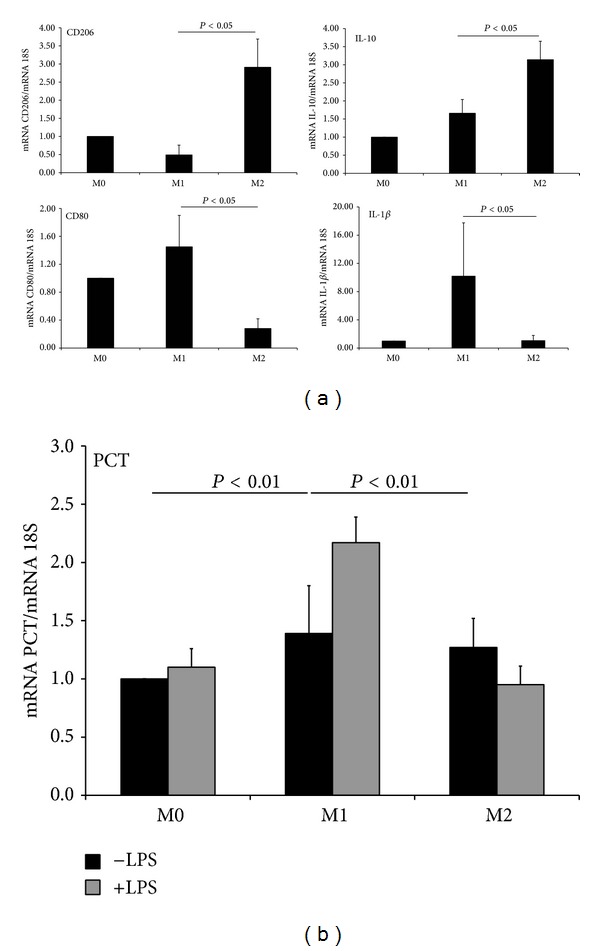
M1 polarized macrophages significantly upregulate the expression of PCT compared to both M0 and M2 macrophages under stimulation with LPS. (a) PMBCs were differentiated in culture for 7 days and polarized O/N with LPS (100 ng/mL) and IFN*γ* (500 U/mL) or IL-4 (20 ng/mL) and IL-10 (50 ng/mL) for the M1/M2 polarization. mRNA expression levels of CD80 and IL-1*β* (M1 markers) and CD206 and IL-10 (M2 markers) were measured by qPCR and normalized to those of the human housekeeping gene 18S. (b) RNA obtained from M0, M1, and M2 macrophages were quantified for PCT and 18S expression by qPCR. The relative amount of mRNA for PCT was normalized with reference to 18S value. Results were expressed as AUs, in which 1 AU represents the value obtained with untreated macrophages used as a positive control. Bars represent the mean ± S.E.M. of at least 3 independent experiments. *P < 0.05 and **P < 0.01 (Mann-Whitney test).

The cells were then polarized into fully differentiated M1 and M2 macrophages subtypes and characterized for the expression of some characteristic markers for the two subpopulations [26]. As shown in Figure 2(a), M1 macrophages showed upregulated gene expression of both the proinflammatory cytokine IL-1*β* and the cell surface marker CD80, while M2 macrophages, compared to M1 polarized or unstimulated macrophages (M0), increased the expression of the anti-inflammatory cytokine IL-10 and the mannose receptor C type 1 (CD206).

To assess if the polarization of the mononuclear phagocytes causes a different production of PCT, macrophages were then skewed towards their M1 and M2 phenotypes and stimulated for 3 hours with LPS to evaluate the modulation of the gene expression of PCT by qPCR. The synthesis of PCT was comparable between nonstimulated macrophages polarized towards their pro- and anti-inflammatory phenotypes compared to M0 macrophages. Indeed, the synthesis increased significantly in macrophages with M1 phenotype stimulated with LPS, compared to both M0 and M2 macrophages stimulated with the same concentration of the stimulus (Figure 2(b)). M1 macrophages were also shown to significantly upregulate (from 4.8 to 6 pg/10^6^ cells) the production of PCT following a stimulation with LPS for 24 hours, compared to those unstimulated with the same phenotype.

### 4.2. Gravid Serum (GS) Induces an M2-Like Phenotype in Cultured Macrophages

**Figure 3:**
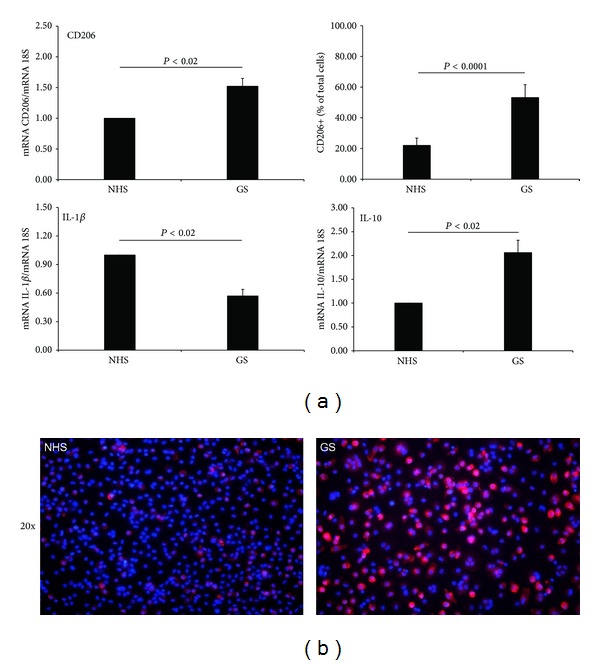
Human macrophages conditioned for 24 hours with gravid serum (GS) downregulate the expression of IL-1*β* and upregulate IL-10. Increased expression of CD206 was observed at gene and protein level. (a) mRNA expression levels of IL-1*β*, IL-10, and CD206 were measured by qPCR and normalized to those of the human housekeeping gene 18S. Results were expressed as AUs, in which 1 AU represents the value obtained with macrophages cultured in NHS used as a positive control. The amount of CD206 positive macrophages was evaluated by counting labeled cells using ImageJ (NIH, United States), considering a percentage of positive cells in at least 5 different fields. Bars represent the mean ± S.E.M. of at least 3 independent experiments. *P < 0.02 and ***P < 0.0001 (Mann-Whitney test). (b) Immunofluorescence reveals the upregulation of the expression of CD206 protein (red) in human macrophages cultured for 24 hours in presence of GS. DAPI nuclear staining is shown in blue. Images were acquired with Leica DM3000 microscope. Original magnification 20x.

We hypothesized that the hormonal changes at the beginning of pregnancy may be able to modulate the phenotype of macrophages. To identify the effects of the first trimester gravid serum (GS) on the macrophage activation we evaluated the gene expression of some pro- and anti-inflammatory markers, modulated during the polarization of macrophages. As shown in Figure 3, we observed that 24 hours of culturing macrophages in the presence of GS significantly overexpressed the CD206 marker both at the gene and protein level. In the same experimental conditions, the expressions of IL-10 and IL-1*β* were, respectively, significantly upregulated and downregulated, meaning that the GS has the ability to skew macrophages through an anti-inflammatory phenotype. Interestingly, we showed that macrophages maintained the ability to respond to proinflammatory stimuli when conditioned with the GS, as they upregulated the expression of the IL-1*β* and TNF*α* cytokines in response to the stimulation with LPS for 3 hours (data not shown).

### 4.3. *In Vitro* Effects of the GS and the Pregnancy Hormones on PCT Expression in Cultured Macrophages

**Figure 4:**
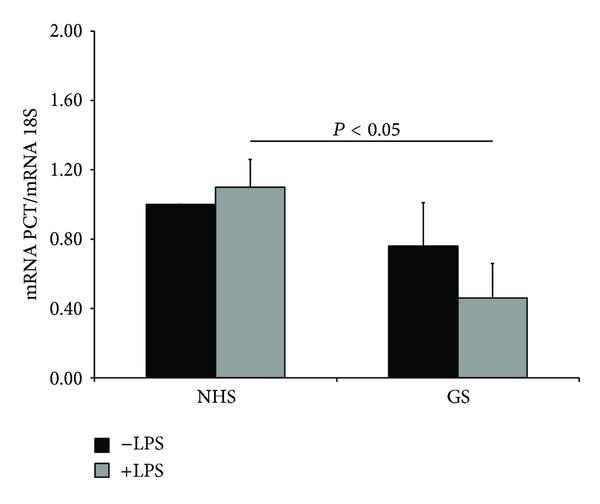
The expression of PCT is negatively regulated in human macrophages conditioned for 24 hours with gravid serum (GS), reaching significance after stimulation with 100 ng/mL of LPS for 3 hours. mRNA expression levels of these proteins were measured by qPCR and normalized to those of the human housekeeping gene 18S. Results were expressed as AUs, in which 1 AU represents the value obtained with macrophages cultured in NHS used as a positive control. Bars represent the mean ± S.E.M. of at least 3 independent experiments. *P < 0.05 (Mann-Whitney test).

Based on the ability of the GS to polarize mononuclear phagocytes through an M2-like phenotype, we evaluated its effect on the expression of PCT. Macrophages cultured for 24 hours in the presence of GS and subsequently stimulated with LPS for 3 hours showed a significant reduction in PCT expression, compared to those cultured in NHS (Figure 4). Notably, the GS itself was able to downregulate the expression of PCT in macrophages during 24 hours of culture, compared to cells cultured in NHS, although the difference was not significant.

**Figure 5:**
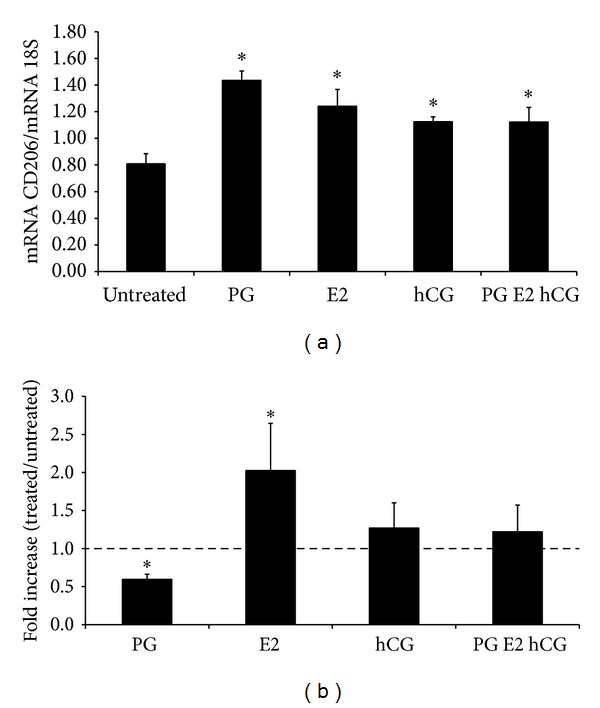
Expression of CD206 and PCT in human macrophages conditioned for 24 hours with PG, E2, and hCG or with the three hormones combined together. (a) The mRNA expression level of CD206 was measured by qPCR and normalized to those of the human housekeeping gene 18S. Results were expressed as AUs, in which 1 AU represents the value obtained with macrophages cultured in NHS used as a positive control. (b) PCT fold increase was expressed as ratio between mRNA levels of LPS stimulated and LPS untreated cells. Bars represent the mean ± S.E.M. of at least 3 independent experiments. *P < 0.05 (Mann-Whitney test).

Furthermore, the hormonal *milieu* of the GS has been taken into account, trying to understand if it could be involved in the polarization of macrophages and if it is able to modulate both PCT and CD206 expression in these cells. Unexpectedly, the stimulation with progesterone (PG) induced a significant downregulation of PCT expression, while we observed an opposite effect of the 17*β*-Estradiol (E2). No modulation of the expression of PCT has been observed in macrophages stimulated with hCG. Interestingly, the three hormones together were not able to modulate PCT expression in macrophages, probably for the opposite effect of PG and E2 (Figure 5(b)). Note that all these hormones shared the ability to polarize macrophages through an M2-like phenotype, as evidenced by the upregulation of the CD206 expression (Figure 5(a)).

## 5. Discussion

This is the first detailed study of PCT production by human macrophages, a powerful biomarker for an early and accurate diagnosis of bacterial infection [1].

The contribution of macrophages to PCT expression and secretion is not clear yet. The only study on the production of PCT by human macrophages in culture is by Linscheid et al. [8], which showed that macrophages at day 5 of culture did not express calcitonin or calcitonin gene-related peptide (CGRP)-I mRNA under basal conditions or after stimulation with several inflammatory mediators. The study demonstrated the ability of PBMCs to secrete PCT only after an adherence to endothelial cells or plastic surfaces. The induction was transient and it was not detectable after 18 hours of culture. Several studies investigated the production of PCT by PBMCs with contrasting results [1]. The presence of PCT has been previously observed by Oberhoffer and colleagues [11] in freshly isolated PBMCs both at transcriptional and translational level. Herget-Rosenthal et al. [27] have also demonstrated that PCT released by PBMCs, isolated from controls and patients with advanced chronic kidney disease, described a correlation between the PCT release from PBMCs and the concentration of PCT in the blood. Moreover, Balog et al. [10] showed that Gram-positive bacteria have the TNF-inducing ability to elevate the intracellular content of PCT in human monocytes, although they hypothesized that other bacterial components can induce PCT directly. Müller et al. [5], in an animal model resembling human sepsis, found out that CT-mRNA was ubiquitously and uniformly expressed in multiple tissues throughout the body in response to sepsis, including peritoneal macrophages.

Our study demonstrates the ability of cultured human macrophages to produce PCT after 7 days of culture. Furthermore, we investigated the effect of LPS in macrophage production of PCT. Our data revealed that human macrophages constitutively produce PCT under basal conditions, but are unable to respond to LPS in terms of PCT expression and production. The main and novel finding of the present study is that the ability to increase the production of PCT after LPS stimulation is acquired when macrophages have been skewed towards their M1 phenotype. On the contrary, no modulation in PCT expression was observed in M2 macrophages stimulated with the same amount of LPS. These data are interesting, since M1 macrophages are generally considered responsible for resistance against intracellular pathogens and are associated with acute bacterial infections and sepsis [18]. Furthermore, based on its structure, it has recently been shown that PCT can interact with bacterial LPS inducing a decrease of its proinflammatory effects [21]. These data indicate that the peculiar ability of M1 to produce PCT is probably associated with their physiological function of defence.

The other important observation of our study is that macrophages cultured in GS, obtained from women during the first trimester of pregnancy, skewed towards an M2-like phenotype and consequently, when stimulated with LPS, significantly decreased the level of mRNA for PCT. In an attempt to identify the factors present in GS serum, able to modulate the expression of PCT in human macrophages, we evaluated the effect of PG, E2, and hCG. Our data showed that hCG has no effect on, while PG downregulates and E2 increases the PCT expression after LPS stimulation. The fact that the combined stimulation with three hormones does not have an impact on the modulation of the expression of PCT indicates that additional factors present in the GS, for example, cytokines such as IL-10 [28], play a role in the regulation of the production of this protein in macrophages. We can speculate that all tissue macrophages change their phenotype getting in contact with serum derived factors during pregnancy. Taking into account that placental-derived components in the GS are more concentrated in the placental *milieu*, we can hypothesize that the phenotype of macrophages obtained in our experiments resembles more those present at the fetal-maternal interface.

Very recent data from Koldehoff et al. [29] demonstrated that the gene profile of monocytes during the first trimester pregnancy differs from the one of monocytes isolated from nonpregnant women. On the basis of these data, we can assume that circulating monocytes may be primed by GS factors too, although this issue needs to be further analyzed.

Pregnancy is associated with a unique immunological condition, characterized by decidual, as well as peripheral immune responses adaptation, in order to guarantee maternal tolerance to the foetus [30]. It has been demonstrated that decidual macrophages express markers of alternative activation, including CD206 and IL-10 [23]. Furthermore, it has been previously shown that soluble circulating factors, present in sera of pregnant women, induce a dendritic cell (DC) incomplete activation that was associated with reduced DC allostimulatory capacity [31]. In this study we demonstrated that during human physiological pregnancy macrophages, in response to human pregnant sera, also undergo profound changes that probably reflect the maternal systemic reaction to the foetus. Our data showed that also the synthesis of PCT is strongly downregulated in macrophages with this particular phenotype, although the response to LPS is conserved in terms of production of the proinflammatory cytokine IL-1*β* (data not shown). These data are in agreement with Mor statements [30], which indicate that the immune system at the implantation site is not suppressed but functionally and carefully controlled. The fact that the expression of PCT is downregulated indicates that it may probably have harmful effects to fetal development and pregnancy progression.

## 6. Conclusion

In conclusion, our results demonstrated that the expression and synthesis of PCT after LPS stimulation are a peculiar ability of M1 macrophages. Furthermore, we found out that macrophages cultured in first trimester GS acquire an M2-like phenotype, not allowing them to produce PCT after LPS stimulation. Based on these data, we can speculate that pregnant women might respond less efficiently to sepsis than nonpregnant women in terms of about 50% reduction of PCT expression in macrophages. Our data, although they were obtained only in an *in vitro* model of cultured human macrophages, indicate that the slowdown of PCT synthesis might have an impact on the use of this laboratory marker for the analysis of the inflammatory status in pregnant women during sepsis and in autoimmune and inflammatory diseases.

Above all, these data support the concept reported by Mor and Cardenas [30] that pregnancy is a unique condition, in which the immune system is modulated, but not suppressed, leading to a differential response against bacterial infections, which is not completely known yet.

## Figures and Tables

**Table 1:** Primer used for qPCR analysis.

Gene	Primers	Sequence 5′→3′	Annealing temperature (°C)	Amplicon size (bp)	Gene bank accession number
18S	For	ATCCCTGAAAAGTTCCAGCA	60	154	NM_022551
	Rev	CCCTCTTGGTGAGGTCAATG			

PCT	For	TCTAAGCGGTGCGGTAATCTG	60	85	NM_001741
	Rev	CAGTTTGGGGGAACGTGTGA			

IL-1*β*	For	TTCCCTGCCCACAGACCTTC	66	298	NM_000576
	Rev	AGGCCCAAGGCCACAGGTAT			

IL-10	For	CCAAGCCTTGTCTGAGATGAT	61	120	NM_000572
	Rev	CTGAGGGTCTTCAGGTTCTCC			

CD80	For	AGGAACACCCTCCAATCTCTG	60	150	NM_005191
	Rev	GGTCAAAAGTGAAAGCCAACA			

CD206	For	TATGGAATAAAGACCCGCTGAC	61	133	NM_002438
	Rev	TGCTCATGTATCTCTGTGATGCT			
